# Corticosteroids for hospitalized patients with mild to critically-ill COVID-19: a multicenter, retrospective, propensity score-matched study

**DOI:** 10.1038/s41598-021-90246-y

**Published:** 2021-05-21

**Authors:** Satoshi Ikeda, Toshihiro Misumi, Shinyu Izumi, Keita Sakamoto, Naoki Nishimura, Shosei Ro, Koichi Fukunaga, Satoshi Okamori, Natsuo Tachikawa, Nobuyuki Miyata, Masaharu Shinkai, Masahiro Shinoda, Yasunari Miyazaki, Yuki Iijima, Takehiro Izumo, Minoru Inomata, Masaki Okamoto, Tomoyoshi Yamaguchi, Keisuke Iwabuchi, Makoto Masuda, Hiroyuki Takoi, Yoshitaka Oyamada, Shigeki Fujitani, Masamichi Mineshita, Haruyuki Ishii, Atsushi Nakagawa, Nobuhiro Yamaguchi, Makoto Hibino, Kenji Tsushima, Tatsuya Nagai, Satoru Ishikawa, Nobuhisa Ishikawa, Yasuhiro Kondoh, Yoshitaka Yamazaki, Kyoko Gocho, Tomotaka Nishizawa, Akifumi Tsuzuku, Kazuma Yagi, Yuichiro Shindo, Yuriko Takeda, Takeharu Yamanaka, Takashi Ogura

**Affiliations:** 1 Department of Respiratory Medicine, Kanagawa Cardiovascular and Respiratory Center, 6-16-1 Tomioka-higashi, Kanazawa-ku, Yokohama-city, Kanagawa 236-0051 Japan; 2 Department of Biostatistics, Yokohama City University School of Medicine, 3-9 Fukuura, Kanazawa-ku, Yokohama-city, Kanagawa 236-0004 Japan; 3 Department of Respiratory Medicine, National Center for Global Health and Medicine, 1-21-1 Toyama, Shinjuku-ku, Tokyo, 162-8655 Japan; 4 Department of Pulmonary Medicine, Thoracic Center, St. Luke’s International Hospital, 9-1 Akashi-cho, Chuo-ku, Tokyo, 104-8560 Japan; 5 Division of Pulmonary Medicine, Department of Medicine, Keio University School of Medicine, 35 Shinanomachi, Shinjuku-ku, Tokyo, 160-8582 Japan; 6 Department of Infectious Disease, Yokohama Municipal Citizen’s Hospital, 1-1 Mitsuzawanishimachi, Kanagawa-ku, Yokohama-city, Kanagawa 221-0855 Japan; 7 Department of Internal Medicine, Tokyo Shinagawa Hospital, 6-3-22 Higashioi, Shinagawa-ku, Tokyo, 140-8522 Japan; 8 Department of Respiratory Medicine, Tokyo Medical & Dental University, 1-5-45 Yushima, Bunkyo-ku, Tokyo, 113-8510 Japan; 9 Department of Respiratory Medicine, Japanese Red Cross Medical Center, 4-1-22 Hiroo, shibuya-ku, Tokyo, 150-8935 Japan; 10 Department of Respirology, Clinical Research Institute, National Hospital Organization Kyushu Medical Center, 1-8-1 Chigyohama, Chuo-ku, Fukuoka, 810-0065 Japan; 11 Department of Respiratory Medicine, Tokyo Rinkai Hospital, 1-4-2 Rinkai-Cho, Edogawa-ku, Tokyo, 123-0086 Japan; 12 Department of General Medicine, Kanagawa Prefectural Ashigarakami Hospital, 866-1 Matsuda-Soryo, Matsuda-machi, Ashigarakami, Kanagawa 258-0003 Japan; 13 Department of Respiratory Medicine, Fujisawa City Hospital, 2-6-1 Fujisawa, Fujisawa-city, Kanagawa 251-8550 Japan; 14 Department of Respiratory Medicine, Tokyo Medical University, 6-7-1 Nishi-Shinjuku, Shinjuku-ku, Tokyo, 160-0023 Japan; 15 Department of Respiratory Medicine, National Hospital Organization Tokyo Medical Center, 2-5-1Meguro-ku, HigashigaokaTokyo, 152-8902 Japan; 16 Department of Emergency and Critical Care Medicine, St Marianna University School of Medicine, 2-16-1 Sugao, Miyamae-ku, Kawasaki-city, Kanagawa 216-8511 Japan; 17 Department of Internal Medicine, Division of Respiratory Medicine, St Marianna University School of Medicine, 2-16-1 Sugao, Miyamae-ku, Kawasaki-city, Kanagawa 216-8511 Japan; 18 Department of Respiratory Medicine, Kyorin University School of Medicine, 6-20-2 Shinkawa, Mitaka-city, Tokyo 181-8611 Japan; 19 Department of Respiratory Medicine, Kobe City Hospital Organization Kobe City Medical Center General Hospital, 2-1-1, Minatojima Minamimachi, Chuo-ku, Kobe-city, Hyogo 650-0047 Japan; 20 Department of Respiratory Medicine, Yokosuka City Hospital, 1-3-2 Nagasaka, Yokosuka-city, Kanagawa 240-0195 Japan; 21 Department of Respiratory Medicine, Shonan Fujisawa Tokushukai Hospital, 1-5-1 Tsujido Kandai, Fujisawa-city, Kanagawa 251-0041 Japan; 22 Department of Pulmonary Medicine, International University of Health and Welfare School of Medicine, 4-3 Kozunomori, Narita-city, Chiba 286-8686 Japan; 23 Department of Emergency and Critical Care Medicine, Tokyo Bay Urayasu-Ichikawa Medical Center, 3-4-32 Todaijima, Urayasu-city, Chiba 279-0001 Japan; 24 Department of Respiratory Medicine, Funabashi Central Hospital, 6 -13-10 Kaijin, Funabashi-city, Chiba 273-8556 Japan; 25 Department of Respiratory Medicine, Hiroshima Prefectural Hospital, 1-5-54 Ujina-kanda, Minami-ku, Hiroshima-city, Hiroshima 734-8530 Japan; 26 Department of Respiratory Medicine and Allergy, Tosei General Hospital, 160 Nishioiwake-cho, Seto-city, Aichi 489-8642 Japan; 27 Center of Infectious Diseases, Nagano Prefectural Shinshu Medical Center, 1332 Suzaka, Suzaka-city, Nagano 382-8577 Japan; 28 Department of Respiratory Medicine, Saiseikai Yokohamashi Tobu Hospital, 3-6-1 Shimosueyoshi, Tsurumi-ku, Yokohama-city, Kanagawa 230-0012 Japan; 29 Department of Respiratory Medicine, Japanese Red Cross Society Saitama Hospital, 1-5 Shintoshin, Chuo-ku, Saitama 330-8553 Japan; 30 Department of Pulmonary Medicine, Gifu Prefectural General Medical Center, 4-6-1 Noisshiki, Gifu-city, Gifu 500-8717 Japan; 31 Department of Pulmpnary Medicine, Keiyu Hospital, 3-7-3 Minatomirai, Nishi-ku, Yokohama-city, Kanagawa 220-8521 Japan; 32 Department of Respiratory Medicine, Nagoya University Graduate School of Medicine, 65 Tsurumai-cho, Showa-ku, Nagoya-city, Aichi 466-8550 Japan

**Keywords:** Clinical microbiology, Virology

## Abstract

Corticosteroids use in coronavirus disease 2019 (COVID-19) is controversial, especially in mild to severe patients who do not require invasive/noninvasive ventilation. Moreover, many factors remain unclear regarding the appropriate use of corticosteroids for COVID-19. In this context, this multicenter, retrospective, propensity score–matched study was launched to evaluate the efficacy of systemic corticosteroid administration for hospitalized patients with COVID-19 ranging in the degree of severity from mild to critically-ill disease. This multicenter, retrospective study enrolled consecutive hospitalized COVID-19 patients diagnosed January–April 2020 across 30 institutions in Japan. Clinical outcomes were compared for COVID-19 patients who received or did not receive corticosteroids, after adjusting for propensity scores. The primary endpoint was the odds ratio (OR) for improvement on a 7-point ordinal score on Day 15. Of 1092 COVID-19 patients analyzed, 118 patients were assigned to either the corticosteroid and non-corticosteroid group, after propensity score matching. At baseline, most patients did not require invasive/noninvasive ventilation (85.6% corticosteroid group vs. 89.8% non-corticosteroid group). The odds of improvement in a 7-point ordinal score on Day 15 was significantly lower for the corticosteroid versus non-corticosteroid group (OR, 0.611; 95% confidence interval [CI], 0.388–0.962; *p* = 0.034). The time to improvement in radiological findings was significantly shorter in the corticosteroid versus non-corticosteroid group (hazard ratio [HR], 1.758; 95% CI, 1.323–2.337; *p* < 0.001), regardless of baseline clinical status. The duration of invasive mechanical ventilation was shorter in corticosteroid versus non-corticosteroid group (HR, 1.466; 95% CI, 0.841–2.554; *p* = 0.177). Of the 106 patients who received methylprednisolone, the duration of invasive mechanical ventilation was significantly shorter in the pulse/semi-pulse versus standard dose group (HR, 2.831; 95% CI, 1.347–5.950; *p* = 0.006). In conclusion, corticosteroids for hospitalized patients with COVID-19 did not improve clinical status on Day 15, but reduced the time to improvement in radiological findings for all patients regardless of disease severity and also reduced the duration of invasive mechanical ventilation in patients who required intubation.

***Trial registration***: This study was registered in the University hospital Medical Information Network Clinical Trials Registry on April 21, 2020 (ID: UMIN000040211).

## Introduction

Patients with coronavirus disease 2019 (COVID-19) have occasionally developed severe pneumonia, and some of these patients progress to life-threatening respiratory failure, acute respiratory distress syndrome (ARDS) and multiple organ failure^1,2^. Although the mechanisms of COVID-19–induced lung injury and multiple organ failure are still being elucidated, patients with severe COVID-19 are reported to have higher serum cytokine levels than those with mild to moderate COVID-19, suggesting that a “cytokine storm” may be one of the etiological factors^2–5^. This condition is associated with rapid deterioration in the severe acute respiratory syndrome coronavirus (SARS-CoV)-1 and Middle East respiratory syndrome coronavirus (MERS-CoV)^6–8^. If the COVID-19–induced lung injury worsens to the degree that invasive mechanical ventilation or extracorporeal membrane oxygen therapy (ECMO) is required, the mortality is very high^9,10^. Therefore, appropriate anti-inflammatory therapy to suppress the cytokine storm is considered crucial to prevent progression to irreversible ARDS and multiple organ failure^11,12^.

Corticosteroid therapy is expected not only to suppress the cytokine storm but also to prevent the progression to pulmonary fibrosis associated with COVID-19 pneumonia, and has been widely used to treat previously prevalent SARS-CoV-1 and MERS-CoV^13,14^. Recent results of several randomized trials of corticosteroids against COVID-19 have been reported^15–18^, in which the therapy reduced the 28-day mortality and increased the number of ventilator-free days in critically ill patients with COVID-19^15,16^. In light of these results, the latest World Health Organization (WHO) guidance recommends corticosteroids for severe and critical patients^19^. On the other hands, most of the randomized trials reported so far did not include non-severe patients who did not require invasive or noninvasive ventilation. Only in the Randomized Evaluation of COVid-19 thERapY (RECOVERY) trial has the efficacy of corticosteroids for non-severe patients been validated, and corticosteroids failed to show a survival benefit for patients not receiving respiratory support, and might even be harmful^15^. Based on this result alone, WHO guidance suggested not to use corticosteroids for the treatment of non-severe patients.

Therefore, the usefulness and necessity of corticosteroids for COVID-19 remains controversial, especially for the patients who do not require invasive or noninvasive ventilation. Moreover, many factors remain unclear regarding the appropriate use of corticosteroids for COVID-19, such as initial dose, administration period, and timing of initiation. In this context, this multicenter, retrospective, propensity score–matched study was launched to evaluate the efficacy of systemic corticosteroid administration for hospitalized patients with COVID-19 ranging in the degree of severity from mild to critically-ill disease. In addition, various subgroup analyses were performed to examine in detail the appropriate use of corticosteroids for COVID-19.

## Methods

### Study design and participants

This multicenter, retrospective study was conducted at 30 institutions in Japan. The study enrolled all consecutive patients who met the following inclusion criteria: (1) SARS-CoV-2 infection confirmed by polymerase chain reaction (PCR) test; (2) diagnosed between January 23–April 30, 2020; (3) required hospitalization for COVID-19; and (4) did not require home oxygen therapy before infection with COVID-19. Clinical and laboratory data were retrieved from patient medical records. Clinical outcomes for COVID-19 patients who received systemic corticosteroids (corticosteroid group) were compared with those who did not receive this therapy (non-corticosteroid group), after adjusting for propensity scores. The case registration period was from May 1–June 30, 2020.

### Propensity score matching

The method of propensity score matching was used to minimize the bias due to confounding factors, assuming that an imbalance in patient background between the corticosteroid and non-corticosteroid groups may exist. The propensity score for each patient was calculated as a probability from a logistic regression model, including all covariates that were considered clinically important and had an impact on the patient's prognosis: (1) gender; (2) age; (3) body mass index; (4) smoking history; (5) comorbid hypertension; (6) comorbid diabetes mellitus; (7) time from symptom onset to admission; (8) score of 7-point ordinal scale on Day 1; (9) oxygen saturation (SpO_2_)/fraction of inspired oxygen (FiO_2_) on Day 1; (10) dyspnea; (11) pneumonia on initial chest X-ray or computed tomography (CT); (12) C-reactive protein (CRP); (13) concomitant use of favipiravir, and (14) concomitant use of any non-steroidal treatment for COVID-19. In mild to severe patients who do not require invasive/noninvasive ventilation, FiO_2_ was estimated from the delivery system and flow rate using a commonly used conversion table.

### Endpoints

The primary endpoint was the odds ratio (OR) for improvement of the score on a 7-point ordinal scale on Day 15, with the first day of hospitalization as Day 1. The ordinal scale is an assessment of the clinical status on a given day. The 7-point scale is as follows: (1) death; (2) hospitalized, on invasive mechanical ventilation or ECMO; (3) hospitalized, on noninvasive positive pressure ventilation (NIPPV) or high-flow nasal cannula (HFNC); (4) hospitalized, requiring low flow supplemental oxygen; (5) hospitalized, not requiring supplemental oxygen, requiring ongoing medical care; (6) hospitalized, not requiring supplemental oxygen, no longer required ongoing medical care; and (7) discharged/not hospitalized.

The key secondary endpoints were as follows: (1) time to PCR negativity of the swab solution; (2) duration of fever; (3) percentage of improvement in radiological findings; (4) time to improvement in radiological findings; (5) proportion of patients requiring invasive mechanical ventilation with tracheal intubation/ECMO; (6) time to requiring invasive mechanical ventilation with tracheal intubation; (7) duration of invasive mechanical ventilation with tracheal intubation; (8) hospitalization period, and (9) survival period.

### Statistical analysis

In the primary analysis, ordinal variables were compared between groups using a proportional odds model. In the secondary and exploratory analysis, time to event was estimated using the Kaplan–Meier method. The Cox proportional hazards model was used to calculate the hazard ratio (HR) and its 95% confidence interval (CI) for the treatment effect between groups. Categorical variables were presented as numbers (percentages), and compared using chi square test or Fisher exact test. Normally distributed continuous variables were presented as mean and standard deviation (SD), and compared using *t* test. Continuous variables related to time were presented as median (interquartile ranges) and compared using *t* test. A *p* value < 0.05 was considered statistically significant. All statistical analyses were performed using statistical software package SAS (version 9.4, SAS Institute).

### Ethics approval and participant consent

This study was performed in accordance with the Declaration of Helsinki. The study protocol was approved by the Ethics Committee of the Kanagawa Cardiovascular and Respiratory Center (approval date: April 21, 2020, approved number: KCRC-20–0004), and the Institutional Review Board or Ethics Committee of other participating facilities. According to the Ethical Guidelines for Medical Research on Human Subjects in Japan, this research falls under the category of research, which does not involve intervention and does not use samples obtained from the human body. The need for patient consent was waived because this was a retrospective study and anonymity was secured. For this reason, the Institutional Review Boards or Ethics Committees of all participating facilities approved that we applied opt-out method by publishing this study on either the participating facility's website or on a bulletin board. This study was registered in the University hospital Medical Information Network Clinical Trials Registry on April 21, 2020 (ID: UMIN000040211).

## Results

### Patient disposition

**Figure 1:**
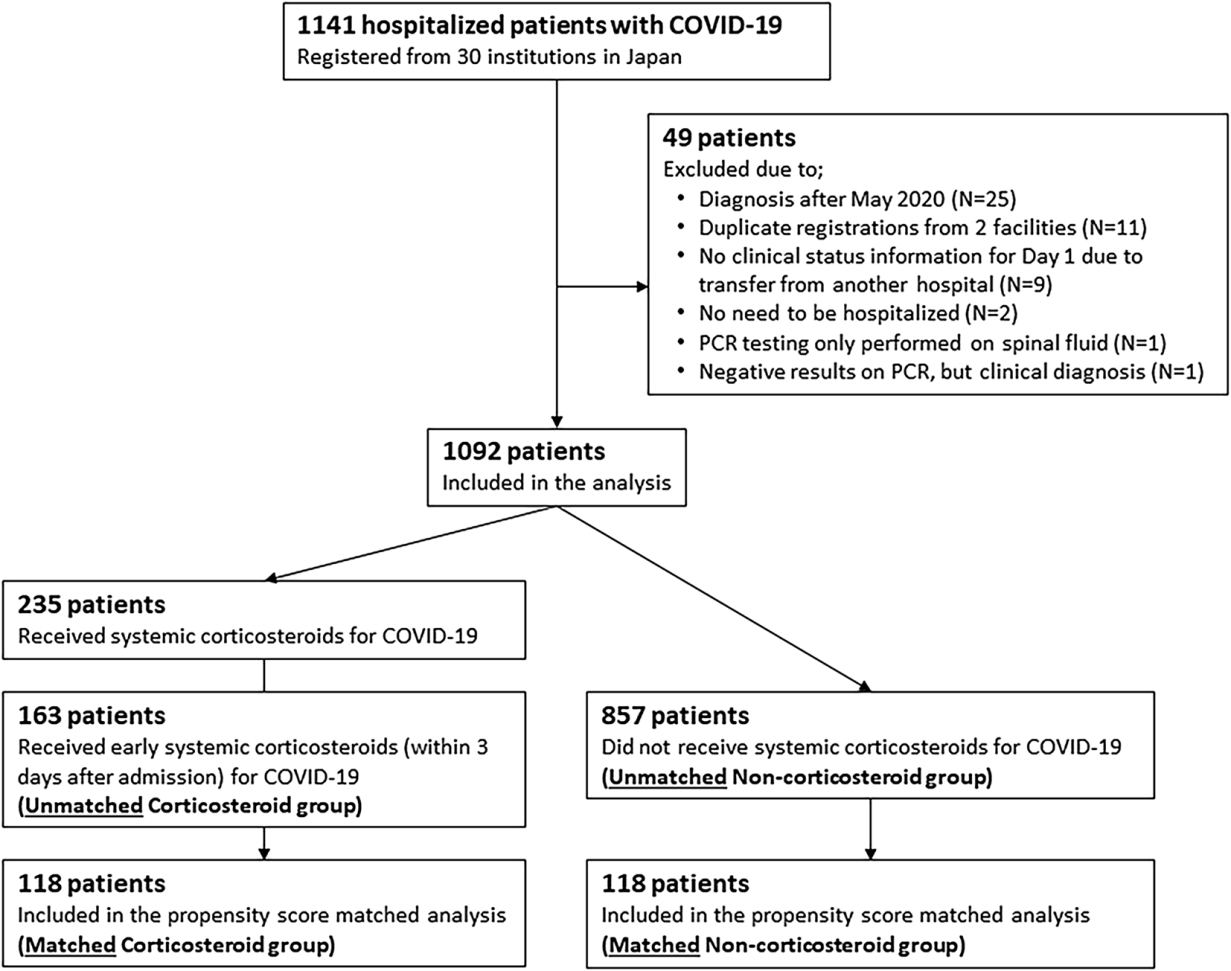
Patient Disposition. COVID-19, coronavirus disease 2019; PCR, polymerase chain reaction.

Of 1141 consecutive hospitalized patients with COVID-19 registered by June 30, 2020, 49 patients were excluded from this study based on these criteria: (1) diagnosis after May 2020 (25 patients); (2) duplicate registrations from 2 facilities (11 patients); (3) no clinical status information for Day 1 due to transfer from another hospital (9 patients); (4) no need to be hospitalized (2 patients), (5) PCR testing only performed on spinal fluid (1 patient), and (6) negative results on PCR, but clinical diagnosis (1 patient). Thus, 1092 patients were included in the final analysis (Fig. 1).

Clinical characteristics and prognosis of the 1092 patients analyzed are shown in Supplemental Table 1 and 2. The mortality was 2.1% on Day 14, and 3.8% on Day 28. Of the 235 patients who received corticosteroids for COVID-19, 163 (69.4%) received early corticosteroids within 3 days after admission. The remaining 72 patients (30.6%) who started corticosteroid > 4 days after admission had a greater decline in SpO_2_/FiO_2_ from admission to just before corticosteroid initiation and a significantly worse score on the 7-point ordinal scale on Day 15 compared with the 163 early-treatment patients (Supplemental Table 3 and 4). We considered that the more patients in the corticosteroid group who started delayed corticosteroids after their respiratory status had already deteriorated, the more difficult it would be to assess the primary endpoint (improvement in clinical status on Day 15) in comparison with the non-corticosteroid group, even using propensity score matching; moreover, early corticosteroids have been reported to be effective against COVID-19.^20^ Therefore, the 163 patients who received early corticosteroids within 3 days after admission were designated as the unmatched corticosteroid group, and the 857 patients who did not receive corticosteroids for COVID-19 were categorized as the unmatched non-corticosteroid group. After propensity score matching, 118 patients were assigned to either the corticosteroid and non-corticosteroid groups.

### Baseline characteristics before/after propensity score matching

The distribution of the patients’ baseline characteristics according to corticosteroid exposure is shown in Table 1, both in the unmatched and matched samples. The unmatched samples included a significantly higher number of male patients and those who were older age, had a higher weight and body mass index, and had more comorbidities (hypertension and diabetes) in the corticosteroid versus non-corticosteroid group. In addition, clinical and laboratory data for the corticosteroid versus non-corticosteroid group showed significantly poorer clinical status in a 7-point ordinal scale on Day 1, lower SpO_2_/FiO_2_, higher rates of fever and dyspnea, higher CRP concentrations, and lower lymphocyte counts.

**Table 1:** Baseline characteristics before and after propensity score matching.

	Unmatched patients			Propensity-Score Matched patients		
	Corticosteroid (N = 163)	Non-corticosteroid (N = 857)	*p*-value	Corticosteroid (N = 118)	Non-corticosteroid (N = 118)	*p*-value
**Gender**—**no. (%)**						
Female	44 (27.0)	322 (37.6)	0.01	34 (28.8)	38 (32.2)	0.572
Male	119 (73.0)	535 (62.4)		84 (71.2)	80 (67.8)	
**Age**—**no. (%)**						
< 40 years	16 (9.8)	253 (29.5)	< 0.0001	15 (12.7)	12 (10.2)	0.885
40–59 years	59 (36.2)	275 (32.1)		44 (37.3)	49 (41.5)	
60–79 years	65 (39.9)	264 (30.8)		44 (37.3)	42 (35.6)	
≥ 80 years	23 (14.1)	65 (7.6)		15 (12.7)	15 (12.7)	
Height—cm	166.5 ± 9.6	166.1 ± 9.8	0.595	166.1 ± 9.5	165.4 ± 9.9	0.57
Body weight—kg	69.9 ± 18.3	66.0 ± 15.5	0.008	69.0 ± 17.9	66.5 ± 13.2	0.231
**Body Mass Index—no. (%)**						
< 18.5	4 (2.8)	55 (7.9)	0.083	3 (2.5)	1 (0.8)	0.584
≥ 18.5, < 25	84 (58.7)	404 (57.9)		70 (59.3)	73 (61.9)	
≥ 25	55 (38.5)	239 (34.2)		45 (38.1)	44 (37.3)	
**Race/region—no. (%)**						
Japanese	158 (96.9)	782 (91.2)	0.129	113 (95.8)	116 (98.3)	0.503
East Asians outside of Japan (China, Korea)	3 (1.8)	15 (1.8)		3 (2.5)	1 (0.8)	
South-East Asians	2 (1.2)	27 (3.2)		2 (1.7)	1 (0.8)	
Westerners*—*Caucasians	0 (0.0)	30 (3.5)				
Westerners*—*Blacks	0 (0.0)	1 (0.1)				
Others	0 (0.0)	2 (0.2)				
**Smoking history—no. (%)**						
Never	91 (59.9)	456 (59.1)	0.854	72 (61.0)	74 (62.7)	0.789
Former or Current	61 (40.1)	316 (40.9)		46 (39.0)	44 (37.3)	
**Comorbidities—no. (%)**						
Hypertension	64 (39.3)	200 (23.3)	< 0.0001	42 (35.6)	37 (31.4)	0.49
Diabetes mellitus	50 (30.7)	118 (13.8)	< 0.0001	33 (28.0)	34 (28.8)	0.885
Time from symptom onset to admission—days	8.4 ± 4.3	8.2 ± 4.8	0.551	8.4 ± 4.4	8.4 ± 3.4	1
**Score of 7-point ordinal scale on Day 1—no. (%)**						
2 or 3	32 (19.6)	21 (2.5)	< 0.0001	15 (12.7)	9 (7.6)	0.537
4	74 (45.4)	168 (19.6)		53 (44.9)	60 (50.8)	
5	54 (33.1)	310 (36.2)		48 (40.7)	46 (39.0)	
6	3 (1.8)	358 (41.8)		2 (1.7)	3 (2.5)	
**SpO2/FiO2**						
On Day 1	342.1 ± 130.5	435.1 ± 70.7	< 0.0001	372.7 ± 112.7	383.3 ± 105.3	0.457
Just before corticosteroid initiation	292.9 ± 139.2			318.8 ± 132.4		
**Symptoms due to COVID-19**						
Fever ≥ 37 °C*—*no. (%)	126 (77.3)	525 (61.3)	< 0.0001	95 (80.5)	86 (72.9)	0.166
Dyspnea—no. (%)	92 (56.4)	249 (29.1)	< 0.0001	63 (53.4)	58 (49.2)	0.515
Taste and/or smell disorder—no. (%)	19 (11.7)	198 (23.1)	0.001	15 (12.7)	25 (21.2)	0.083
Pneumonia on initial Xp/CT—no. (%)	159 (97.5)	600 (70.0)	< 0.0001	116 (98.3)	116 (98.3)	1
**Laboratory data**						
C-reactive protein—mg/dL	9.7 ± 7.1	4.4 ± 5.7	< 0.0001	8.6 ± 6.9	8.3 ± 7.5	0.748
Lymphocyte count—/µL	861.8 ± 592.2	1193.9 ± 588.2	< 0.0001	848.2 ± 646.6	1005.8 ± 589.1	0.062

Unmatched patients refer to the total of 1020 enrolled patients (163 patients who received early corticosteroids for COVID-19 and 857 patients who did not receive systemic corticosteroids for COVID-19) subject to propensity score matching in this study. Because only a few patients had a baseline 7-point ordinal score of 3, the patients with a baseline score of 2 and 3 were combined for the analysis. Categorical variables were presented as numbers (%), and compared using the chi square test. Normally distributed continuous variables were presented as mean and standard deviation (SD), and compared using the *t* test. A *p* value of < 0.05 was considered statistically significant. COVID-19, coronavirus disease 2019; CT, computed tomography.

**Figure 2:**
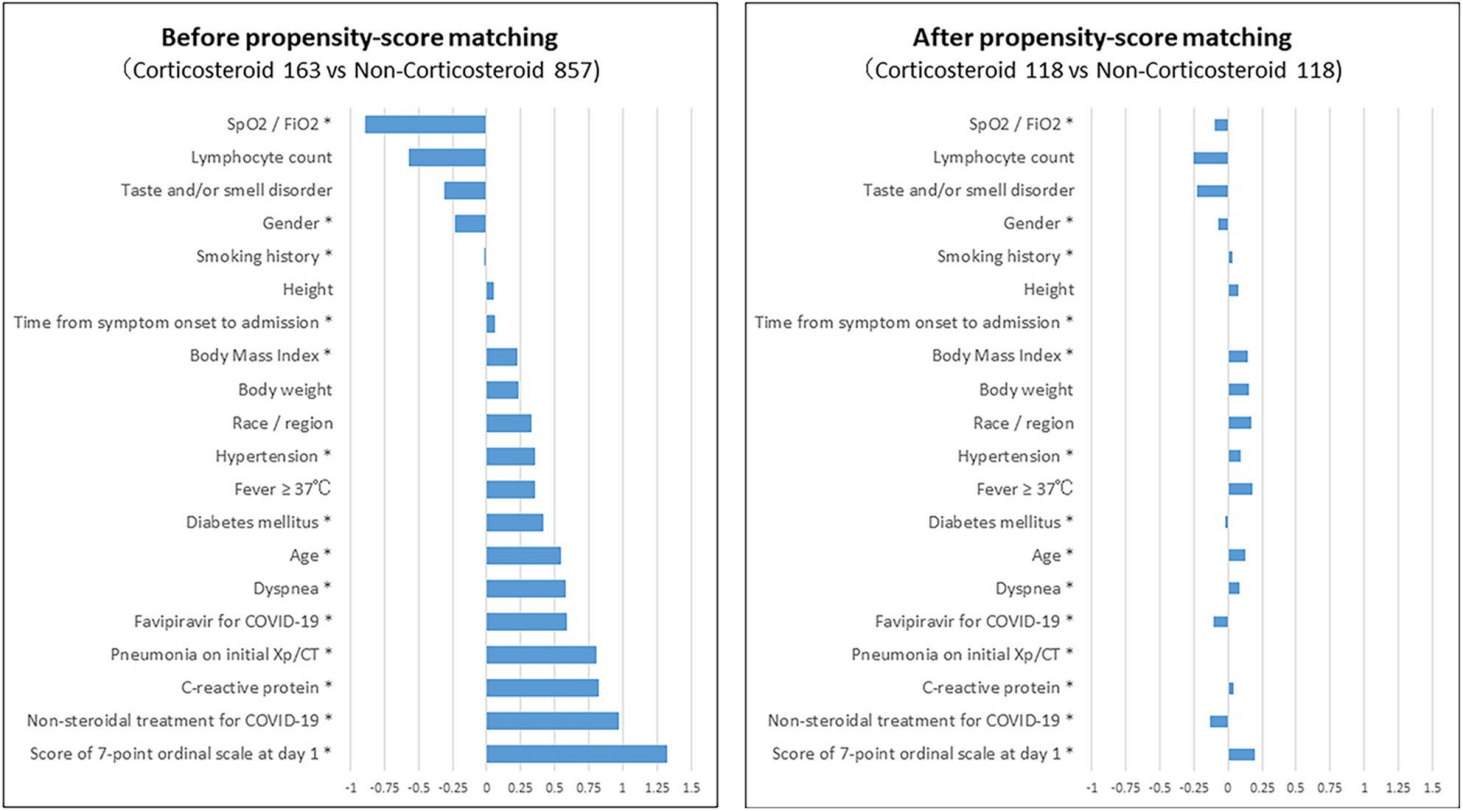
Standardized Mean Difference Before and After Propensity Score Matching. * Covariates used for propensity score matching. COVID-19, coronavirus disease 2019; CT, computed tomography.

Standardized mean differences for each covariate before and after propensity score matching are shown in Fig. 2. The differences between corticosteroid and pretreatment variables were attenuated in the matched versus unmatched samples for propensity score. In fact, baseline characteristics were well balanced between the corticosteroid and non-corticosteroid groups after propensity score matching (Table 1). Regarding the baseline score on the 7-point ordinal scale in the matched samples, 4 was the most common score for both the corticosteroid and non-corticosteroid groups (44.9% vs. 50.8%), followed by a score of 5 (40.7% vs. 39.0%).

Regarding the specific COVID-19 treatment administered both in the propensity score–unmatched and matched corticosteroid groups, nearly 90% of the corticosteroids administered for COVID-19 were methylprednisolone, with a median starting dose of 80 mg/day and a mean administration period of 11.0 days (Table2).

**Table 2:** Treatment for coronavirus disease 2019.

	Unmatched patients			Propensity-score matched patients		
	Corticosteroid (N = 163)	Non-corticosteroid (N = 857)	*p*-value	Corticosteroid (N = 118)	Non-corticosteroid (N = 118)	*p*-value
**Corticosteroids for COVID-19**						
Methylprednisolone	144 (88.3)	–		106 (89.8)	–	
Starting dose						
Median—mg/day	80	–		80	–	
Minimum–Maximum—mg/day	12–1000	–		12–1000	–	
Duration of administration—days	11.0 [5.0, 16.0]	–		11.0 [6.0, 15.8]	–	
Oral prednisolone	7 (4.3)	–		5 (4.2)	–	
Starting dose						
Median—mg/day	40	–		40	–	
Minimum–Maximum—mg/day	30–80	–		30–55	–	
Duration of administration—days	15.0 [12.5, 17.5]	–		15.0 [15.0, 18.0]	–	
Dexamethasone	6 (3.7)	–		1 (0.8%)	–	
Starting dose						
Median—mg/day	16	–		80	–	
Minimum–Maximum—mg/day	8–80	–		–	–	
Duration of administration—days	9.0 [8.0, 10.0]	–		20.0	–	
Others—no. (%)	6 (3.7)	–		6 (5.1%)	–	
**Non-steroidal treatment for COVID-19**						
None—no. (%)	1 (0.6)	286 (33.4)	< 0.0001	1 (0.8)	0	0.316
Favipiravir—no. (%)	98 (60.1)	275 (32.1)	< 0.0001	73 (61.9)	79 (66.9)	0.415
Lopinavir/ritonavir—no. (%)	6 (3.7)	48 (5.6)	0.316	5 (4.2)	11 (9.3)	0.12
Chloroquine—no. (%)	33 (20.2)	108 (12.6)	0.01	14 (11.9)	18 (15.3)	0.447
Ciclesonide—no. (%)	35 (21.5)	201 (23.5)	0.582	22 (18.6)	41 (34.7)	0.005
Tocilizumab—no. (%)	6 (3.7)	8 (0.9)	0.006	3 (2.5)	1 (0.8)	0.313
Macrolide—no. (%)	114 (69.9)	215 (25.1)	< 0.0001	81 (68.6)	36 (30.5)	< 0.0001
Immunoglobulin—no. (%)	9 (5.5)	14 (1.6)	0.002	8 (6.8)	6 (5.1)	0.582
Others—no. (%)	102 (62.6)	166 (19.4)	< 0.0001	80 (67.8)	30 (25.4)	< 0.0001

Unmatched patients refer to the total of 1020 enrolled patients (163 patients who received early corticosteroids for COVID-19 and 857 patients who did not receive systemic corticosteroids for COVID-19) subject to propensity score matching in this study. Categorical variables were presented as numbers (%), and compared using the chi square test. Continuous variables related to time were presented as median [interquartile ranges] and compared using the *t* test. A *p* value of < 0.05 was considered statistically significant. COVID-19, coronavirus disease 2019.

### Primary outcome

The odds of improvement in a 7-point ordinal scale on Day 15 were significantly lower in the corticosteroid versus non-corticosteroid group (OR, 0.611; 95% CI, 0.388–0.962; *p* = 0.034) (Table 3). In critically ill patients with a baseline 7-point ordinal score of 2 or 3, the clinical status on Day 15 was similar in both groups (OR, 0.953; 95% CI, 0.215–4.224; *p* = 0.950). In contrast, for patients with mild to severe disease with a baseline score of 4 or 5, the odds of improvement were lower in the corticosteroid group than in the non-corticosteroid group.

**Table 3:** Primary outcome.

	Overall		Score of 7-point ordinal scale on Day 1 (baseline)					
			2,3		4		5	
	Corticosteroid	Non-corticosteroid	Corticosteroid	Non-corticosteroid	Corticosteroid	Non-corticosteroid	Corticosteroid	Non-corticosteroid
	(N = 118)	(N = 118)	(N = 15)	(N = 9)	(N = 53)	(N = 60)	(N = 48)	(N = 46)
**Score of 7-point ordinal scale on Day 15—no. (%)**								
1	7 (5.9)	3 (2.5)	2 (13.3)	0	3 (5.7)	3 (5.0)	2 (4.2)	0
2	16 (13.6)	12 (10.2)	2 (13.3)	3 (33.3)	10 (18.9)	9 (15.0)	3 (6.3)	0
3	4 (3.4)	1 (0.8)	1 (6.7)	0	2 (3.8)	1 (1.7)	1 (2.1)	0
4	29 (24.6)	22 (18.6)	5 (33.3)	4 (44.4)	19 (35.8)	16 (26.7)	4 (8.3)	1 (2.2)
5	20 (16.9)	27 (22.9)	3 (20.0)	0	4 (7.5)	10 (16.7)	13 (27.1)	16 (34.8)
6	16 (13.6)	21 (17.8)	2 (13.3)	2 (22.2)	10 (18.9)	11 (18.3)	4 (8.3)	7 (15.2)
7	26 (22.0)	32 (27.1)	0	0	5 (9.4)	10 (16.7)	21 (43.8)	22 (47.8)
Odds ratio (95%CI)	0.611 (0.388–0.962)		0.953 (0.215–4.224)		0.626 (0.323–1.213)		0.589 (0.277–1.255)	
*p*-value	0.034		0.950		0.165		0.170	

Because only a few patients had a baseline 7-point ordinal score of 3, the patients with a baseline score of 2 and 3 were combined for the analysis. Categorical variables were presented as numbers (%). Ordinal variables were compared between groups using a proportional odds model. A *p* value of < 0.05 was considered statistically significant. CI, confidence interval.

### Key secondary outcomes

**Figure 3:**
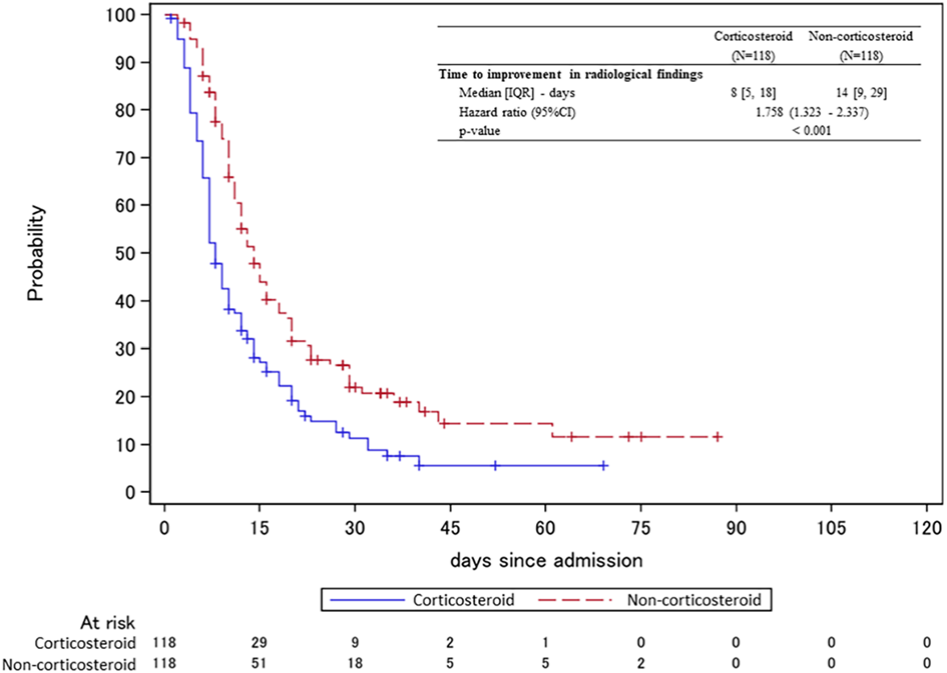
Time to Improvement in Radiological Findings. Kaplan–Meier curves for the time to improvement in radiological findings. Cox proportional hazards model was used to calculate the hazard ratio and its 95% confidence interval for the treatment effect between groups. IQR, interquartile ranges; CI, confidence interval.

**Figure 4:**
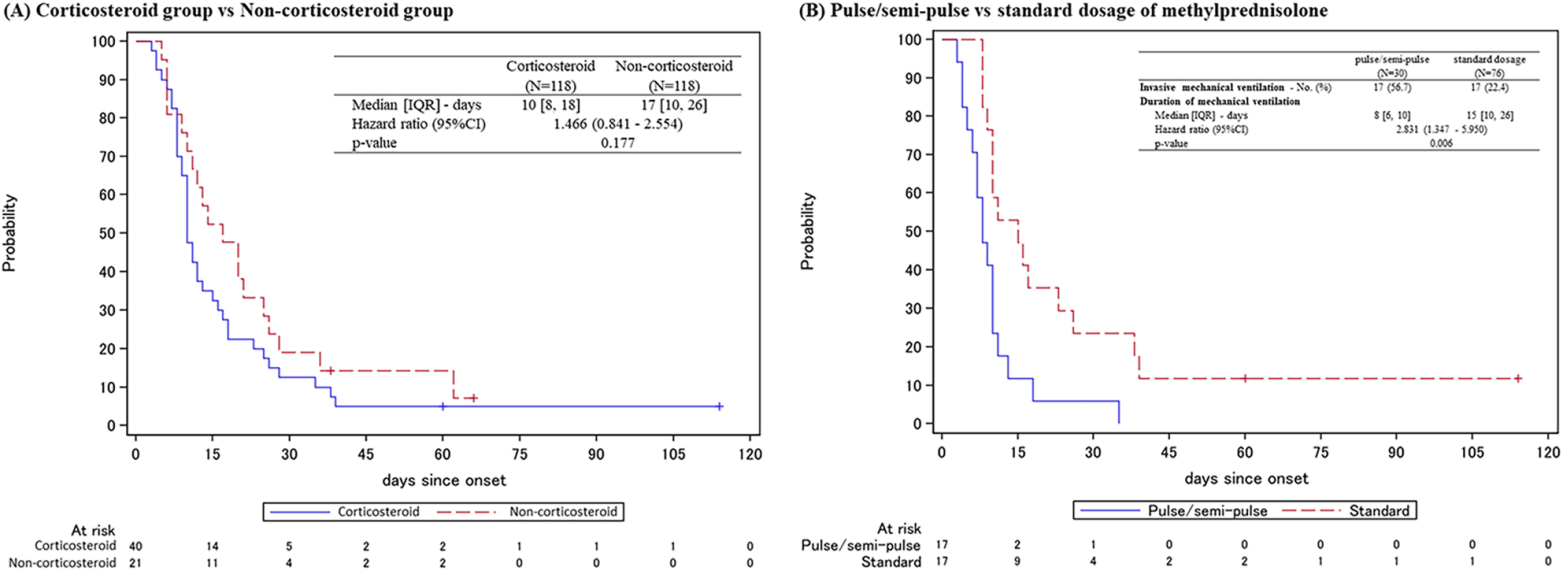
Duration of Invasive Mechanical Ventilation. (**A**) Kaplan–Meier curves for the duration of invasive mechanical ventilation comparing corticosteroid group and non-corticosteroid group. (**B**) Kaplan–Meier curves for the duration of invasive mechanical ventilation comparing the pulse/semi-pulse group (initial dose ≥ 250 mg/day) and the standard dose group (initial dose < 250 mg/day) among patients receiving methylprednisolone. Cox proportional hazards model was used to calculate the hazard ratio and its 95% confidence interval for the treatment effect between groups. IQR, interquartile ranges; CI, confidence interval.

The key secondary outcomes are shown in Table 4. No significant differences were observed between the two groups with respect to time to PCR negativity or duration of hospitalization. The duration of fever was significantly longer in the corticosteroid group (HR, 0.746; 95% CI, 0.560–0.994; *p* = 0.045). The time to improvement in radiological findings was significantly shorter in the corticosteroid versus non-corticosteroid group (HR, 1.758; 95% CI, 1.323–2.337; *p* < 0.001), regardless of baseline score of 7-point ordinal scale (Fig. 3). The number of patients requiring invasive mechanical ventilation was higher in the corticosteroid versus non-corticosteroid group (33.9% vs. 17.8%; *p* = 0.0072), with median time from admission to tracheal intubation of 2 days for both groups (Supplemental Fig. 1). The duration of invasive mechanical ventilation was shorter in the corticosteroid versus non-corticosteroid group (HR, 1.466; 95% CI, 0.841–2.554; *p* = 0.177) (Fig. 4A). Mortality on Day 28 tended to be higher in the corticosteroid versus non-corticosteroid group (10.2% vs. 4.2%; *p* = 0.1289), and the HR was 2.417 (95% CI, 0.868–6.733; *p* = 0.091) (Supplemental Fig. 2A).

**Table 4:** Secondary outcomes.

	Overall		Score of 7-point ordinal scale on Day 1 (baseline)					
			2,3		4		5	
	Corticosteroid	Non-corticosteroid	Corticosteroid	Non-corticosteroid	Corticosteroid	Non-corticosteroid	Corticosteroid	Non-corticosteroid
	(N = 118)	(N = 118)	(N = 15)	(N = 9)	(N = 53)	(N = 60)	(N = 48)	(N = 46)
**Time to PCR negativity of the swab solution**								
Median [IQR]—days	19 [10, 24]	18 [12, 24]	21 [15, 26]	46 [23, –]	21 [15, 27]	19 [13, 28]	13 [8, 23]	16 [11, 20]
Hazard ratio (95%CI)	1.091 (0.828–1.437)		3.008 (0.948–9.543)		0.908 (0.608–1.356)		1.146 (0.743–1.766)	
*p*-value	0.535		0.062		0.637		0.538	
**Duration of fever**								
Median [IQR]—days	8.5 [4, 16]	6 [4, 12]	7.5 [5, 28]	9.5 [3.5, 18.5]	7.5 [4, 14]	9 [5, 20]	10 [6, 30]	5 [3, 7]
Hazard ratio (95%CI)	0.746 (0.560–0.994)		0.765 (0.289–2.022)		1.231 (0.823–1.843)		0.251 (0.147–0.428)	
*p*-value	0.045		0.589		0.312		< 0.001	
Improvement in radiological findings—No. (%)	103 (87.3)	90 (76.3)	12 (80.0)	6 (66.7)	45 (84.9)	42 (70.0)	44 (91.7)	39 (84.8)
**Time to improvement in radiological findings**								
Median [IQR]*—*days	8 [5, 18]	14 [9, 29]	6.5 [4, 12]	26 [15, 61]	10 [6, 23]	14 [10, 36]	7 [5, 13]	11 [7, 22]
Hazard ratio (95%CI)	1.758 (1.323–2.337)		3.812 (1.285–11.303)		1.541 (1.012–2.349)		1.86 (1.193–2.901)	
*p*-value	< 0.001		0.016		0.044		0.006	
Invasive mechanical ventilation—No. (%)	40 (33.9)	21 (17.8)	11 (73.3)	9 (100)	23 (43.4)	12 (20)	5 (10.4)	0
Extra-Corporeal Membrane Oxygenation—No. (%)	7 (5.9)	7 (5.9)	1 (6.7)	2 (22.2)	5 (9.4)	5 (8.3)	1 (2.1)	0
**Duration of invasive mechanical ventilation**								
Median [IQR]*—*days	10 [8, 18]	17 [10, 26]	8 [6, 12]	12 [6, 17]	10 [9, 25]	20.5 [12, 31]		
Hazard ratio (95%CI)	1.466 (0.841–2.554)		1.808 (0.691–4.730)		1.642 (0.773–3.489)		–	
*p*-value	0.177		0.227		0.197		–	
**Hospitalization period**								
Median [IQR]—days	24 [15, 34]	21 [14, 29]	27 [20, 33]	27 [26, 34]	30 [20, 47]	23 [17, 37]	16.5 [11, 26]	16.5 [12, 24]
Hazard ratio (95%CI)	0.861 (0.659–1.125)		1.623 (0.649–4.054)		0.789 (0.530–1.176)		0.797 (0.523–1.216)	
*p*-value	0.272		0.300		0.244		0.293	
**Mortality—No. (%)**								
On day 14	7 (5.9)	3 (2.5)	2 (13.3)	0	3 (5.7)	3 (5.0)	2 (4.2)	0
*p*-value	0.333		0.511		1.000		0.495	
On day 28	12 (10.2)	5 (4.2)	3 (20.0)	1 (11.1)	6 (11.3)	4 (6.7)	3 (6.3)	0
*p*-value	0.129		1.000		0.512		0.242	
During the entire observation period	14 (11.9)	5 (4.2)	3 (20.0)	1 (11.1)	7 (13.2)	4 (6.7)	4 (8.3)	0
**Survival period**								
Hazard ratio (95%CI)	2.417 (0.868–6.733)		1.900 (0.198–18.273)		1.744 (0.509–5.969)		–	
*p*-value	0.091		0.578		0.376		0	

Because only a few patients had a baseline 7-point ordinal score of 3, the patients with a baseline score of 2 and 3 were combined for the analysis. Categorical variables were presented as numbers (%). IQR, interquartile ranges; CI, confidence interval; PCR, polymerase chain reaction.

### Subgroup analysis based on initial dose, administration period and timing of corticosteroids

Subgroup analysis was performed based on initial dose, administration period, and timing of corticosteroids (Table 5). Of the 106 patients who received methylprednisolone, the duration of invasive mechanical ventilation was significantly shorter in the pulse/semi-pulse group (initial dose ≥ 250 mg/day) than in the standard dose group (initial dose < 250 mg/day) (median, 8 days vs. 15 days; HR, 2.831; 95% CI, 1.347–5.950; *p* = 0.006) (Fig. 4B). In the patients receiving corticosteroids for ≤ 10 days, the time to PCR negativity of the swab solution tended to be shorter (HR, 1.437; 95% CI, 0.968–2.132; *p* = 0.072) compared with the patients receiving corticosteroids for > 11 days.

**Table 5:** Subgroup analysis in the propensity-score matched corticosteroid group.

	Initial dose of methylprednisolone (N = 106)		Administration period (N = 118)		Timing of corticosteroids initiation (N = 118)	
	Pulse/semi-pulse	Standard dose	≤ 10 days	> 11 days	On day 1	on day 2 or 3
	(N = 30)	(N = 76)	(N = 55)	(N = 63)	(N = 70)	(N = 48)
**Time to PCR negativity of the swab solution**						
Median [IQR]—days	22 [15, 24]	15 [9, 24]	15 [8, 24]	21 [14, 26]	15 [9, 24]	20 [15, 27]
Hazard ratio (95%CI)	0.854 (0.537–1.358)		1.437 (0.968–2.132)		0.753 (0.504–1.124)	
*p*-value	0.505		0.072		0.165	
**Duration of fever**						
Median [IQR]—days	8 [4, 16]	10 [5, 19]	6 [4, 15]	11 [6, 28]	8 [4, 15]	10 [4, 19]
Hazard ratio (95%CI)	1.037 (0.639–0.1.683)		1.337 (0.886–2.019)		0.927 (0.610–1.407)	
*p*-value	0.882		0.167		0.720	
**Time to improvement in radiological findings**						
Median [IQR]—days	11 [4, 23]	8 [6, 16]	7 [5, 14]	9 [6, 18]	7 [5, 16]	8.5 [6, 18]
Hazard ratio (95%CI)	0.880 (0.554–1.399)		1.124 (0.760–1.662)		0.855 (0.576–1.271)	
*p*-value	0.589		0.557		0.439	
*Invasive mechanical ventilation—No. (%)*	17 (56.7)	17 (22.4)	9 (16.4)	31 (49.2)	18 (25.7)	22 (45.8)
**Duration of invasive mechanical ventilation**						
Median [IQR]*—*days	8 [6, 10]	15 [10, 26]	10 [8, 16]	11 [8, 18]	10 [7, 13]	11.5 [9, 23]
Hazard ratio (95%CI)	2.831 (1.347–5.950)		1.247 (0.588–2.646)		0.713 (0.374–1.358)	
*p*-value	0.006		0.565		0.304	
**Mortality—No. (%)**						
On day 14	2 (6.7)	5 (6.6)	5 (9.1)	2 (3.2)	3 (4.3)	4 (8.3)
On day 28	4 (13.3)	6 (7.9)	6 (10.9)	6 (9.5)	6 (8.6)	6 (12.5)
During the entire observation period	4 (13.3)	8 (10.5)	7 (12.7)	7 (11.1)	6 (8.6)	8 (16.7)

Categorical variables were presented as numbers (percentages). Continuous variables related to time were presented as median [interquartile ranges]. Cox proportional hazards model was used to calculate the hazard ratio and its 95% confidence interval for the treatment effect between groups. A *p* value of < 0.05 was considered statistically significant. IQR, interquartile ranges; CI, confidence interval; PCR, polymerase chain reaction.

### Safety outcome

Safety outcomes for both the corticosteroid and non-corticosteroid groups were also analyzed. Results showed no significant difference in the frequency of thromboembolism between the corticosteroid and non-corticosteroid groups (2.5% vs. 3.4%).

## Discussion

This study demonstrated the following 3 important clinical observations. First, corticosteroids did not lead to avoidance of tracheal intubation or lower mortality in patients with mild to severe COVID-19. Second, for the critically ill patients, corticosteroid therapy reduced not only the time to improvement in radiological findings, but also the duration of invasive mechanical ventilation. Third, methylprednisolone pulse/semi-pulse therapy significantly shortened the duration of invasive mechanical ventilation compared with the standard dose.

In both the corticosteroid and non-corticosteroid groups, > 85% of patients did not require invasive or noninvasive ventilation at baseline. Because data are lacking on the benefit of corticosteroids for patients with mild to severe COVD-19 at baseline, the results of this study deserve to be noteworthy. In mild to severe patients with a baseline 7-point ordinal score of 4 or 5, the clinical status on Day 15 tended to be worse in the corticosteroid group than in the non-corticosteroid group. Administration of corticosteroids to patients with mild to severe COVD-19 was expected to prevent the progression to critical conditions that would require ECMO or invasive mechanical ventilation. However, a high proportion of patients in the corticosteroid group required invasive mechanical ventilation, despite the fact that corticosteroids reduced the time to improvement in radiological findings and suggested some benefit. This finding may have been due to the fact that the time from admission to tracheal intubation in patients who required invasive mechanical ventilation (median 2 days) was shorter than the time to improvement in imaging findings (median 8 days). Because corticosteroid treatment takes a certain amount of time to show benefit, it may not be expected to improve short-term outcomes, such as the avoidance of tracheal intubation.

This study also suggested that corticosteroids may have a negative impact on survival as assessed by Day 14, Day 28 mortality, and the HR in mild to severe patients with a baseline 7-point ordinal score of 4 or 5 (Supplemental Fig. 2C and 2D). Similarly, the RECOVERY study suggested that dexamethasone may rather worsen the prognosis among patients who were not receiving any respiratory support at randomization^15^. Although there is concern that corticosteroid administration within 7 days of onset may inhibit antibody production, the median time from symptom onset to admission in the corticosteroid group in this study was 8.4 days. One possible explanation is that adverse effects of corticosteroids may have affected the prognosis. In the present study, no increase in thromboembolism was observed with corticosteroids. Although it cannot be ruled out that impaired hyperglycemic control and secondary infections may have an impact on prognosis, these data were not collected in this study and are a limitation for the application of study findings. However, it is questionable whether such events really have a significant impact on prognosis. Among the studies of COVID-19 patients requiring hospitalization and treatment, the patient population included in this study had a clearly better prognosis with a lower mortality than that described in many previous reports^21,22^. Although the cause is unclear, the mortality is low not only in Japan, but also in most of the countries in East and Southeast Asia^23^. In light of these findings, it may be difficult to draw definitive conclusions about the survival endpoint based on the results of this study, and caution should be used when comparing and interpreting data from this study with data from previous studies of populations in Europe and in North and South America.

Meanwhile, for the critically ill patients with a baseline ordinal score of 2 or 3, clinical status on Day 15 assessed by the 7-point ordinal scale was similar between the corticosteroid and non-corticosteroid groups. The present study showed 2 positive effects of corticosteroids as expected for critically ill COVID-19 patients. First, the reduction in the time to improvement in radiological findings may suggest the effectiveness of corticosteroids. Second, corticosteroids tended to reduce the duration of invasive mechanical ventilation, which is consistent with previous reports from randomized clinical trials. In the CoDEX trial, dexamethasone significantly increased the number of ventilator-free days in patients with moderate to severe ARDS who required intubation and ventilation^17^. Although this study had a relatively small proportion of critical ill cases at baseline, early administration of corticosteroids to patients who develop severe respiratory failure requiring invasive or noninvasive ventilation may be beneficial.

It is also noteworthy that a subgroup analysis in this study showed that methylprednisolone pulse/semi-pulse therapy shortened the duration of mechanical intubation compared with the usual dose regimen. A small, single-blind, randomized, controlled, clinical trial in Iran reported that methylprednisolone pulse therapy (intravenous injection, 250 mg/day for 3 days) reduced the time of clinical improvement and discharge from the hospital or death in severe hospitalized patients compared to the standard of care^24^. However, to date, no previous reports have examined the differences in efficacy of different starting dose of corticosteroids. In contrast, subgroup analysis by administration period in this study suggested that prolonged corticosteroid administration over 11 days may prolong the time to PCR negativity. This result suggests that prolonged corticosteroid administration may delay the elimination of the virus from the body. Therefore, high-dose, short-term corticosteroid therapy should be considered in critically ill patients with COVID-19 pneumonia.

As a limitation of this study, even among the groups matched for propensity score, the corticosteroid group may still have included more rapidly deteriorating patients than the non-corticosteroid group. In fact, a higher rate of invasive mechanical ventilation (many of which cases are introduced within a few days), longer-lasting fevers despite corticosteroid therapy, and worsening of SpO_2_/FiO_2_ even in a short period of time from baseline to just before corticosteroid initiation were observed in the matched corticosteroid group. As a possible reason, although the impact on severity and prognosis of COVID-19 patients differed greatly among the covariates used in the propensity score matching method in this study, all of these covariates were treated as having equal weight. In addition, the presence or absence of each variable implies the equivalence between multiple variables in terms of severity, but this is not appropriate because being dyspneic is clearly not the same as being diabetic, at least for COVID-19. Therefore, the methodology used for matching was one of the limitations of this study. The biomarker to identify a rapidly deteriorating population among COVID-19 is not well established and may have been difficult, at least with the factors used for propensity score matching in this study. Although serum ferritin concentration could not be used for matching in this study because of the large number of deficiencies, this variable may be useful as a biomarker (serum ferritin concentration was measured for only 26 patients in the corticosteroid group [mean 1534.9 μg/dL] and 51 in the non-corticosteroid group [mean 774.1 μg/dL]). As for radiological findings, it was difficult to make a uniform and detailed evaluation because some patients did not have CT scans, so we only collected information on the presence of pneumonia as determined by the investigator. Detailed analysis of baseline CT images may also be a useful biomarker based on reports that the degree of extension of lung opacities and lung volume loss on CT had an impact on prognosis^25–28^. Because the clinical data in this study were collected retrospectively from the electronic medical records of each 30 participating institutions, and because we were afraid to overburden the investigators in the current situation where COVID-19 is still raging, subjective symptoms such as dyspnea and comorbidities such as diabetes were assessed only for presence or absence and not for severity of illness. As an additional limitation, it is necessary to discuss whether the endpoint using an ordinal scale was valid as a primary endpoint. None of the previously reported studies evaluating the efficacy of corticosteroids for COVID-19 have met the endpoint on an ordinal scale^17^. Many infectious disease studies have relatively short-term measures set as primary endpoints, but the primary endpoint for the study of COVID-19 may need to be established with a longer-term perspective.

## Conclusions

Corticosteroids for hospitalized patients with COVID-19 did not improve clinical status on Day 15. However, corticosteroids reduced not only the time to improvement in radiological findings in all patients regardless of disease severity, but also the duration of invasive mechanical ventilation in the critically ill patients. Methylprednisolone pulse/semi-pulse therapy significantly shortened the duration of invasive mechanical ventilation compared with the standard dose.

## Supplementary Information

Supplementary Information 1.

Supplementary Information 2.

Supplementary Information 3.

## Data Availability

The datasets generated during and/or analyzed during the current study are available from the corresponding author on reasonable request.

## Abbreviations

ARDS: Acute respiratory distress syndrome
CI: Confidence interval
COVID-19: Coronavirus disease 2019
CRP: C-reactive protein
CT: Computed tomography
ECMO: Extracorporeal membrane oxygen therapy
WHO: World Health Organization
FiO_2_: Fraction of inspired oxygen
HFNC: High-flow nasal cannula
HR: Hazard ratio
IL: Interleukin
MERS-CoV: Middle East respiratory syndrome coronavirus
NIPPV: Noninvasive positive pressure ventilation
OR: Odds ratio
PCR: Polymerase chain reaction
SARS-CoV: Severe acute respiratory syndrome coronavirus
SD: Standard deviation
SpO_2_: Oxygen saturation

## References

[1] Cao X (2020). COVID-19: immunopathology and its implications for therapy. Nat. Rev. Immunol..

[2] Huang C, Wang Y, Li X (2020). Clinical features of patients infected with 2019 novel coronavirus in Wuhan, China. Lancet.

[3] Chen N, Zhou M, Dong X (2020). Epidemiological and clinical characteristics of 99 cases of 2019 novel coronavirus pneumonia in Wuhan, China: a descriptive study. Lancet.

[4] Mehta P, McAuley DF, Brown M (2020). COVID-19: consider cytokine storm syndromes and immunosuppression. Lancet.

[5] Bao J, Li C, Zhang K, Kang H, Chen W, Gu B (2020). Comparative analysis of laboratory indexes of severe and non-severe patients infected with COVID-19. Clin. Chim. Acta.

[6] de Wit E, van Doremalen N, Falzarano D, Munster VJ (2016). SARS and MERS: recent insights into emerging coronaviruses. Nat. Rev. Microbiol..

[7] Huang KJ, Su IJ, Theron M (2005). An interferon-gamma-related cytokine storm in SARS patients. J. Med. Virol..

[8] Zhou J, Chu H, Li C (2014). Active replication of Middle East respiratory syndrome coronavirus and aberrant induction of inflammatory cytokines and chemokines in human macrophages: Implications for pathogenesis. J. Infect. Dis..

[9] Wu C, Chen X, Cai Y (2020). Risk factors associated with acute respiratory distress syndrome and death in patients with coronavirus disease 2019 pneumonia in Wuhan, China. JAMA Intern. Med..

[10] Qin, C., Zhou, L., Hu, Z., *et al*. Dysregulation of immune response in patients with COVID-19 in Wuhan, China. *Clin. Infect. Dis.* ciaa248 (2020). 10.1093/cid/ciaa248 PMC7108125 32161940

[11] McGonagle D, Sharif K, O’Regan A, Bridgewood C (2020). The role of cytokines including interleukin-6 in COVID-19 induced pneumonia and macrophage activation syndrome-like disease. Autoimmun. Rev..

[12] Hirano T, Murakami M (2020). COVID-19: A new virus, but a familiar receptor and cytokine release syndrome. Immunity.

[13] Stockman LJ, Bellamy R, Garner P (2006). SARS: systematic review of treatment effects. PLoS Med..

[14] Arabi YM, Mandourah Y, Al-Hameed F (2018). corticosteroid therapy for critically ill patients with middle east respiratory syndrome. Am. J. Respir. Crit. Care Med..

[15] RECOVERY Collaborative Group, Horby, P., Lim, W. S., *et al*. Dexamethasone in hospitalized patients with Covid-19—preliminary report. *N. Engl. J. Med.* NEJMoa2021436 (2020). [Online ahead of print].

[16] Writing Committee for the REMAP-CAP Investigators, Angus, D. C., Derde, L., *et al*. Effect of hydrocortisone on mortality and organ support in patients with severe COVID-19: the REMAP-CAP COVID-19 corticosteroid domain randomized clinical trial. *JAMA* (2020). [Online ahead of print] 10.1001/jama.2020.17022 PMC7489418 32876697

[17] Tomazini, B. M., Maia, I. S., Cavalcanti, A. B., *et al*. Effect of dexamethasone on days alive and ventilator-free in patients with moderate or severe acute respiratory distress syndrome and COVID-19: the CoDEX Randomized Clinical Trial. *JAMA* (2020). [Online ahead of print] 10.1001/jama.2020.17021 PMC7489411 32876695

[18] Dequin, P. F., Heming, N., Meziani, F., *et al*. Effect of hydrocortisone on 21-day mortality or respiratory support among critically ill patients with COVID-19: a randomized clinical trial. *JAMA* (2020). [Online ahead of print] 10.1001/jama.2020.16761 PMC7489432 32876689

[19] World Health Organization. Corticosteroids for COVID-19: Living guidance 2 September 2020. https://www.who.int/publications-detail-redirect/WHO-2019-nCoV-Corticosteroids-2020.1. Accessed 14 September 2020 [Internet].

[20] Fadel, R., Morrison, A. R., Vahia, A., *et al*. Early short course corticosteroids in hospitalized patients with COVID-19. *Clin. Infect. Dis.* (2020). [Online ahead of print] 10.1093/cid/ciaa601 PMC7314133 32427279

[21] Wiersinga WJ, Rhodes A, Cheng AC, Peacock SJ, Prescott HC (2020). Pathophysiology, transmission, diagnosis, and treatment of coronavirus disease 2019 (COVID-19): a review. JAMA.

[22] Richardson S, Hirsch JS, Narasimhan M (2020). Presenting characteristics, comorbidities, and outcomes among 5700 patients hospitalized with COVID-19 in the New York City Area. JAMA.

[23] Johns Hopkins University & Medicine. Coronavirus Resource Center. https://coronavirus.jhu.edu/data/mortality. Accessed 5 October 2020 [Internet].

[24] Edalatifard, M., Akhtari, M., Salehi, M., *et al*. Intravenous methylprednisolone pulse as a treatment for hospitalised severe COVID-19 patients: results from a randomised controlled clinical trial. *Eur. Respir. J.* (2020). [Online ahead of print]. 10.1183/13993003.02808-2020 PMC7758541 32943404

[25] Colombi D, Bodini FC, Petrini M (2020). Well-aerated lung on admitting chest CT to predict adverse outcome in COVID-19 pneumonia. Radiology.

[26] Liu F, Zhang Q, Huang C (2020). CT quantification of pneumonia lesions in early days predicts progression to severe illness in a cohort of COVID-19 patients. Theranostics.

[27] Wang Y, Chen Y, Wei Y (2020). Quantitative analysis of chest CT imaging findings with the risk of ARDS in COVID-19 patients: a preliminary study. Ann. Transl. Med..

[28] Iwasawa T, Sato M, Yamaya T (2020). Ultra-high-resolution computed tomography can demonstrate alveolar collapse in novel coronavirus (COVID-19) pneumonia. Jpn. J. Radiol..

