# Supplementary figures and images for: Corticosteroids for hospitalized patients with mild to critically-ill COVID-19: a multicenter, retrospective, propensity score-matched study
Source: Sci Rep. 2021 May 21;11:10727. doi: 10.1038/s41598-021-90246-y (PMC8140087; doi:10.1038/s41598-021-90246-y)

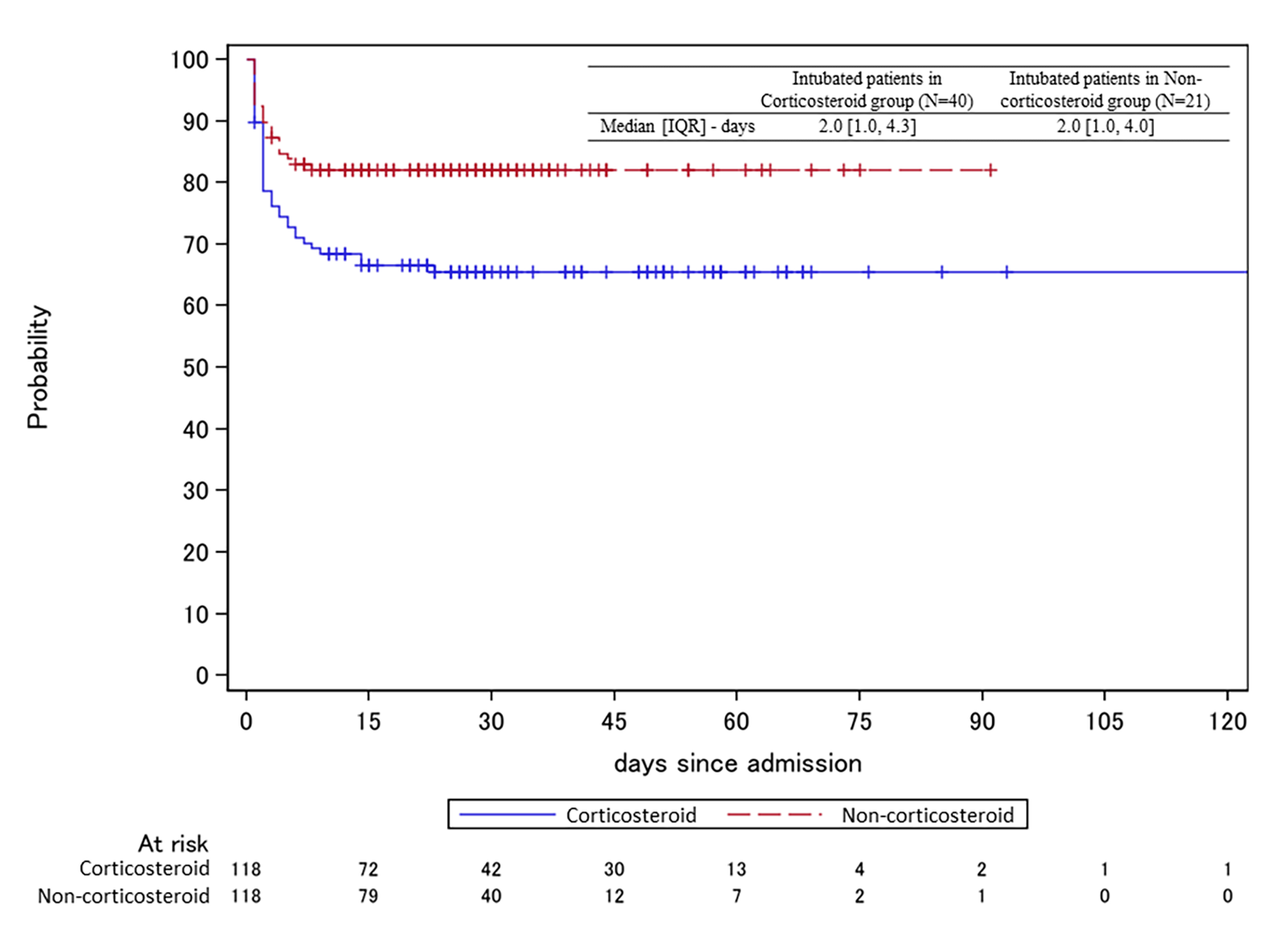

Supplement: Supplementary file 1 — Supplementary Information 1. [file 41598_2021_90246_MOESM1_ESM.tif]

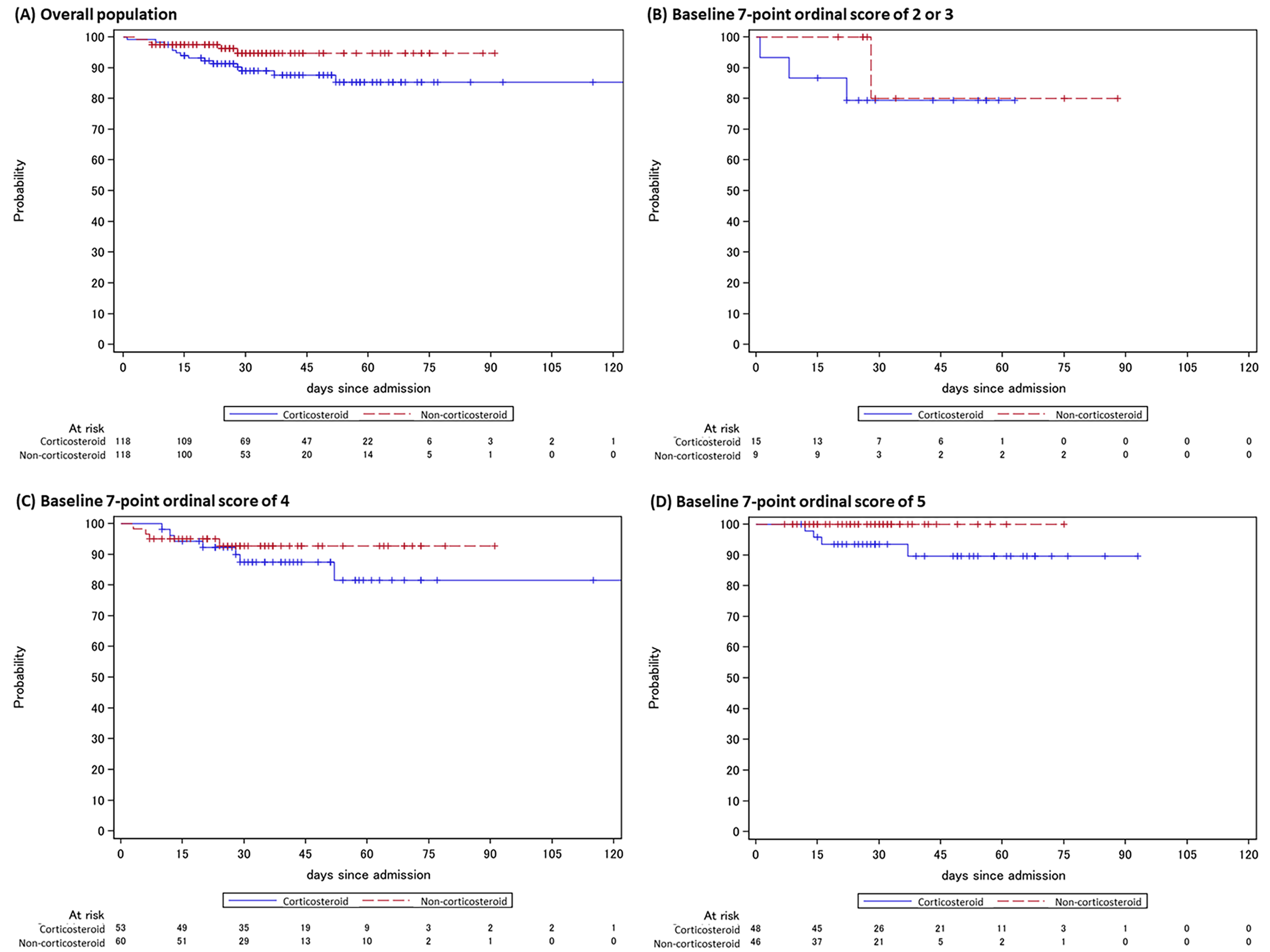

Supplement: Supplementary file 2 — Supplementary Information 2. [file 41598_2021_90246_MOESM2_ESM.tif]
