# Supplementary material for: Corticosteroids for hospitalized patients with mild to critically-ill COVID-19: a multicenter, retrospective, propensity score-matched study
Source: Sci Rep. 2021 May 21;11:10727. doi: 10.1038/s41598-021-90246-y (PMC8140087; doi:10.1038/s41598-021-90246-y)
Supplement: Supplementary file 3 — Supplementary Information 3. [file 41598_2021_90246_MOESM3_ESM.docx]

**Corticosteroids for hospitalized patients with mild to critically-ill COVID-19: A multicenter, retrospective, propensity score–matched study**

Satoshi Ikeda, Toshihiro Misumi, Shinyu Izumi, Keita Sakamoto, Naoki Nishimura, Shosei Ro, Koichi Fukunaga, Satoshi Okamori, Natsuo Tachikawa, Nobuyuki Miyata, Masaharu Shinkai, Masahiro Shinoda, Yasunari Miyazaki, Yuki Iijima, Takehiro Izumo, Minoru Inomata, Masaki Okamoto, Tomoyoshi Yamaguchi, Keisuke Iwabuchi, Makoto Masuda, Hiroyuki Takoi, Yoshitaka Oyamada, Shigeki Fujitani, Masamichi Mineshita, Haruyuki Ishii, Atsushi Nakagawa, Nobuhiro Yamaguchi, Makoto Hibino, Kenji Tsushima, Tatsuya Nagai, Satoru Ishikawa, Nobuhisa Ishikawa, Yasuhiro Kondoh, Yoshitaka Yamazaki, Kyoko Gocho, Tomotaka Nishizawa, Akifumi Tsuzuku, Kazuma Yagi, Yuichiro Shindo, Yuriko Takeda, Takeharu Yamanaka, and Takashi Ogura

**SUPPLEMENTAL MATERIALS**

**Supplemental Table 1. Baseline Characteristics of the Overall Study Population**

| **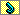** |  | **(N = 1092)** |
| --- | --- | --- |
| **Gender** - no. (%) | |  |
|  | female | 381 (34.9) |
|  | male | 711 (65.1) |
| **Age** - no. (%) | |  |
|  | < 40 yr | 271 (24.8) |
|  | 40 - 59 yr | 357 (32.7) |
|  | 60 - 79 yr | 362 (33.2) |
|  | ≥ 80 yr | 102 (9.3) |
| **Height** - cm | | 166.3±9.8 |
| **Body weight** - kg | | 67.0±16.0 |
| **Body Mass Index** - no. (%) | |  |
|  | < 18.5 | 62 (6.9) |
|  | ≥ 18.5, < 25 | 514 (56.9) |
|  | ≥ 25 | 327 (36.2) |
| **Race/region** - no. (%) | |  |
|  | Japanese | 1008 (92.3) |
|  | East Asians outside of Japan (China, Korea) | 20 (1.8) |
|  | South-East Asians | 30 (2.7) |
|  | Westerners - Caucasians | 31 (2.8) |
|  | Westerners - Blacks | 1 (0.1) |
|  | Others | 2 (0.2) |
| **Smoking history** - no. (%) | |  |
|  | Never | 571 (57.9) |
|  | Former or Current | 416 (42.1) |
| **Comorbidities** - no. (%) | |  |
|  | Hypertension | 299 (27.4) |
|  | Diabetes mellitus | 194 (17.8) |
| **Time from symptom onset to admission** - days | | 8.1±4.7 |
| **Score of 7-point ordinal scale on Day 1** - no. (%) | |  |
|  | 2 or 3 | 62 (5.7) |
|  | 4 | 278 (25.5) |
|  | 5 | 385 (35.3) |
|  | 6 | 367 (33.6) |
| **SpO_2_ / FiO_2_ on Day1** | | 416.4±92.6 |
| **Symptoms due to COVID-19** | |  |
|  | Fever ≥ 37℃ - no. (%) | 714 (65.4) |
|  | Dyspnea - no. (%) | 378 (34.6) |
|  | Taste and/or smell disorder - no. (%) | 227 (20.8) |
| **Pneumonia on initial Xp/CT** - no. (%) | | 827 (75.7) |
| **Laboratory data** | |  |
|  | C-reactive protein - mg/dL | 5.5±6.4 |
|  | Lymphocyte count - /µL | 1135.7±651.3 |
| **Treatment for COVID-19** | | |
|  | Favipiravir - no. (%) | 421 (38.6) |
|  | Macrolide - no. (%) | 367 (33.6) |
|  | Ciclesonide - no. (%) | 264 (24.2) |
|  | Corticosteroid - no. (%) | 235 (21.5) |
|  | Chloroquine - no. (%) | 161 (14.7) |
|  | Lopinavir/ritonavir - no. (%) | 70 (6.4) |
|  | Immunoglobulin - no. (%) | 32 (2.9) |
|  | Tocilizumab - no. (%) | 19 (1.7) |
|  | Others - no. (%) | 306 (28.0) |

Because only a few patients had a baseline 7-point ordinal score of 3, the patients with a baseline score of 2 and 3 were combined for the analysis. Categorical variables were presented as numbers (%), and compared using the chi square test. Normally distributed continuous variables were presented as mean and standard deviation (SD), and compared using the *t* test. A *p* value of <0.05 was considered statistically significant. COVID-19, coronavirus disease 2019; CT, computed tomography.

**Supplemental Table 2. Outcomes for the Overall Study Population**

|  |  | (N=1092) |
| --- | --- | --- |
| Score of 7-point ordinal scale on Day 15 - no. (%) | |  |
|  | 1 (death) | 23 (2.1) |
|  | 2 (hospitalized, on invasive mechanical ventilation or Extra-Corporeal Membrane Oxygenation) | 96 (8.8) |
|  | 3 (hospitalized, on Non-invasive Positive Pressure Ventilation or High-Flow Nasal Cannula) | 10 (0.9) |
|  | 4 (hospitalized, requiring low flow supplemental oxygen) | 116 (10.6) |
|  | 5 (hospitalized, not requiring supplemental oxygen, requiring ongoing medical care) | 140 (12.8) |
|  | 6 (hospitalized, not requiring supplemental oxygen, no longer required ongoing medical care) | 287 (26.3) |
|  | 7 (discharged/not hospitalized) | 401 (36.7) |
|  | unknown | 19 (1.7) |
| Time to PCR negativity of the swab solution - days | | 15 [10, 23] |
| Duration of fever - days | | 7 [4, 13] |
| Oxygen supplementation - no. (%) | | 472 (43.3) |
| Non-invasive Positive Pressure Ventilation or High-Flow Nasal Cannula - no. (%) | | 39 (3.6) |
| Invasive mechanical ventilation with tracheal intubation - no. (%) | | 172 (15.8) |
| Extra-Corporeal Membrane Oxygenation - no. (%) | | 38 (3.5) |
| Hospitalization period - days | | 18 [12, 27] |
| Mortality - no. (%) | |  |
|  | on Day 14 | 23 (2.1) |
|  | on Day 28 | 41 (3.8) |
|  | during the entire observation period | 59 (5.4) |

Categorical variables were presented as numbers (%). Continuous variables related to time were presented as median [interquartile ranges]. PCR, polymerase chain reaction.

**Supplemental Table 3. Baseline Characteristics of the Patients who Received Corticosteroids**

| **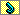** |  | **All patients receiving corticosteroids (N=235)** | **Within 3 days after admission (N = 163)** | **≥ 4 days after admission (N = 72)** | *p*-value |
| --- | --- | --- | --- | --- | --- |
| **Gender** - no. (%) | |  |  |  |  |
|  | female | 59 (25.1) | 44 (27.0) | 15 (20.8) | 0.315 |
|  | male | 176 (74.9) | 119 (73.0) | 57 (79.2) |  |
| **Age** - no. (%) | |  |  |  |  |
|  | < 40 yr | 18 (7.7) | 16 (9.8) | 2 (2.8) | 0.186 |
|  | 40 - 59 yr | 82 (34.9) | 59 (36.2) | 23 (31.9) |  |
|  | 60 - 79 yr | 98 (41.7) | 65 (39.9) | 33 (45.8) |  |
|  | ≥ 80 yr | 37 (15.7) | 23 (14.1) | 14 (19.4) |  |
| **Height** - cm | | 167.0±9.6 | 166.5±9.6 | 168.2±9.5 | 0.247 |
| **Body weight** - kg | | 70.4±17.1 | 69.9±18.3 | 71.5±14.2 | 0.524 |
| **Body Mass Index** - no. (%) | |  |  |  |  |
|  | < 18.5 | 7 (3.4) | 4 (2.8) | 3 (4.8) | 0.082 |
|  | ≥ 18.5, < 25 | 110 (53.7) | 84 (58.7) | 26 (41.9) |  |
|  | ≥ 25 | 88 (42.9) | 55 (38.5) | 33 (53.2) |  |
| **Race/region** - no. (%) | |  |  |  |  |
|  | Japanese | 226 (96.2) | 158 (96.9) | 68 (94.4) | 0.473 |
|  | East Asians outside of Japan (China, Korea) | 5 (2.1) | 3 (1.8) | 2 (2.8) |  |
|  | South-East Asians | 3 (1.3) | 2 (1.2) | 1 (1.4) |  |
|  | Westerners - Caucasians | 1 (0.4) | 0 (0.0) | 1 (1.4) |  |
| **Smoking history** - no. (%) | |  |  |  |  |
|  | Never | 115 (53.5) | 91 (59.9) | 24 (38.1) | 0.004 |
|  | Former or Current | 100 (46.5) | 61 (40.1) | 39 (61.9) |  |
| **Comorbidities** - no. (%) | |  |  |  |  |
|  | Hypertension | 99 (42.1) | 64 (39.3) | 35 (48.6) | 0.181 |
|  | Diabetes mellitus | 76 (32.3) | 50 (30.7) | 26 (36.1) | 0.412 |
| **Time from symptom onset to admission** - days | | 8.0±4.1 | 8.4±4.3 | 7.0±3.6 | 0.018 |
| **Score of 7-point ordinal scale on day 1** - no. (%) | |  |  |  |  |
|  | 2 or 3 | 41 (17.4) | 32 (19.6) | 9 (12.5) | 0.060 |
|  | 4 | 110 (46.8) | 74 (45.4) | 36 (50.0) |  |
|  | 5 | 75 (31.9) | 54 (33.1) | 21 (29.2) |  |
|  | 6 | 9 (3.8) | 3 (1.8) | 6 (8.3) |  |
| **SpO2 / FiO2** | |  |  |  |  |
|  | at day 1 | 349.0±125.4 | 342.1±130.5 | 364.8±112.2 | 0.204 |
|  | just before corticosteroid initiation | 273.6±134.7 | 292.8±139.1 | 230.4±113.6 | 0.001 |
| **Symptoms due to COVID-19** | |  |  |  |  |
|  | Fever ≥ 37℃ - no. (%) | 189 (80.4) | 126 (77.3) | 63 (87.5) | 0.069 |
|  | Dyspnea - no. (%) | 129 (54.9) | 92 (56.4) | 37 (51.4) | 0.473 |
|  | Taste and/or smell disorder - no. (%) | 29 (12.4) | 19 (11.7) | 10 (14.1) | 0.604 |
| **Pneumonia on initial Xp/CT** - no. (%) | | 227 (96.6) | 159 (97.5) | 68 (94.4) | 0.227 |
| **Laboratory data** | |  |  |  |  |
|  | C-reactive protein - mg/dL |  |  |  |  |
|  | at day 1 | 9.6±7.1 | 9.7±7.1 | 9.4±7.1 | 0.803 |
|  | just before corticosteroid initiation | 11.8±8.3 | 10.2±7.5 | 15.3±9.1 | <0.001 |
|  | Lymphocyte count - /µL | 919.4±811.5 | 861.8±592.2 | 1048.6±1157.9 | 0.120 |
| **Treatment for COVID-19** | | | | | |
|  | Favipiravir - no. (%) | 146 (62.1) | 98 (60.1) | 48 (66.7) | 0.340 |
|  | Macrolide - no. (%) | 152 (64.7) | 114 (69.9) | 38 (52.8) | 0.011 |
|  | Ciclesonide - no. (%) | 63 (26.8) | 35 (21.5) | 28 (38.9) | 0.006 |
|  | Chloroquine - no. (%) | 53 (22.6) | 33 (20.2) | 20 (27.8) | 0.203 |
|  | Lopinavir/ritonavir - no. (%) | 22 (9.4) | 6 (3.7) | 16 (22.2) | <0.001 |
|  | Immunoglobulin - no. (%) | 18 (7.7) | 9 (5.5) | 9 (12.5) | 0.064 |
|  | Tocilizumab - no. (%) | 11 (4.7) | 6 (3.7) | 5 (6.9) | 0.275 |
|  | Others - no. (%) | 140 (59.6) | 102 (62.6) | 38 (52.8) | 0.158 |

Because only a few patients had a baseline 7-point ordinal score of 3, the patients with a baseline score of 2 and 3 were combined for the analysis. Categorical variables were presented as numbers (%), and compared using the chi square test. Normally distributed continuous variables were presented as mean and standard deviation (SD), and compared using the *t* test. A *p* value of <0.05 was considered statistically significant. COVID-19, coronavirus disease 2019; CT, computed tomography.

**Supplemental Table 4. Outcome of the Patients who Received Corticosteroids**

|  |  | Within 3 days after admission (N = 163) | ≥ 4 days after admission (N = 72) | Odds ratio or Hazard ratio (95%CI) | *p*-value |
| --- | --- | --- | --- | --- | --- |
| Score of 7-point ordinal scale on Day 15 - no. (%) | |  |  |  |  |
|  | 1 | 10 (6.1) | 5 (6.9) | 0.597 (0.363-0.982) | 0.042 |
|  | 2 | 28 (17.2) | 17 (23.6) |  |  |
|  | 3 | 5 (3.1) | 3 (4.2) |  |  |
|  | 4 | 39 (23.9) | 20 (27.8) |  |  |
|  | 5 | 25 (15.3) | 12 (16.7) |  |  |
|  | 6 | 26 (16.0) | 9 (12.5) |  |  |
|  | 7 | 28 (17.2) | 5 (6.9) |  |  |
|  | unknown | 2 (1.2) | 1 (1.4) |  |  |
| Time to PCR negativity of the swab solution - days | | 20 [13, 25] | 21 [16, 30] | 1.479 (1.073-2.039) | 0.017 |
| Duration of fever - days | | 10 [4, 19] | 11 [7, 16] | 0.949 (0.702-1.285) | 0.737 |
| Invasive mechanical ventilation - no. (%) | | 62 (38.0) | 32 (44.4) | - | 0.355 |
| Duration of invasive mechanical ventilation - days | | 10.5 [8, 26] | 14.5 [8.5, 26] | 1.007 (0.648-1.565) | 0.975 |
| Hospitalization period - days | | 26 [16, 37] | 27 [19, 36] | 1.057 (0.785-1.424) | 0.715 |
| Mortality - no. (%) | |  |  |  |  |
|  | on Day 14 | 10 (6.1) | 5 (6.9) | - | 0.779 |
|  | on Day 28 | 18 (11.0) | 10 (13.9) | - | 0.520 |
|  | during the entire observation period | 23 (14.1) | 14 (19.4) | - | 0.333 |

Categorical variables were presented as numbers (%). Continuous variables related to time were presented as median [interquartile ranges]. Ordinal variables were compared between groups using a proportional odds model. Cox proportional hazards model was used to calculate the hazard ratio and its 95% confidence interval for the treatment effect between groups. A *p* value of <0.05 was considered statistically significant. CI, confidence interval; PCR, polymerase chain reaction.

**Supplemental Figure 1. Time from Admission to Tracheal Intubation**

Kaplan–Meier curves for the time from admission to tracheal intubation. IQR, interquartile ranges.

**Supplemental Figure 2. Survival Curve**

Kaplan–Meier curves for survival in the overall population (A), baseline 7-point ordinal score of 2 or 3 (B), baseline score of 4 (C), and baseline score of 5 (D). Because only a few patients had a baseline score of 3, the patients with a baseline score of 2 and 3 were combined for the analysis.
